# Supplementary material for: The diversity of floral temperature patterns, and their use by pollinators
Source: eLife. 2017 Dec 19;6:e31262. doi: 10.7554/eLife.31262 (PMC5736352; doi:10.7554/eLife.31262)
Supplement: Supplementary file 1: — Species are ordered taxonomically. The temperature at the hottest and coldest region of the flower and the difference in temperature between these points is also given. Plants derived from the same species where counted together for occurrence or average temperature difference calculations. Plants not used in the calculations are marked with a ‘*’ next to their Δ temp. value. [file elife-31262-supp1.docx]

**Supplementary File 1:** A summary of the temperature patterns observed on each of the 118 species thermographed, and the additional 18 cultivars and subspecies. Species are ordered taxonomically. The temperature at the hottest and coldest region of the flower and the difference in temperature between these points is also given. Plants derived from the same species where counted together for occurrence or average temperature difference calculations. Plants not used in the calculations are marked with a ‘*’ next to their Δ temp. value.

| Plant species | Order | Family | Floral Symmetry (Radial/Bilateral) | Time | Date | Location | Hot region temp. ºC | Cold region temp. ºC | Δ temp. ºC |
| --- | --- | --- | --- | --- | --- | --- | --- | --- | --- |
| *Anthurium scherzerianum* | Alismarales | Araceae | Bilateral | 14:37 | 26/06/2014 | Bristol | 24.8 | 23.3 | 1.5 |
| *Zantedeschia aethiopica* | Alismarales | Araceae | Bilateral | 14:58 | 26/06/2014 | Bristol | 20.9 | 19.9 | 1 |
| *Astrantia major* | Apiales | Apiaceae | Radial | 11:22 | 26/06/2014 | Bristol | 19.2 | 18.7 | 0.5 |
| *Allium cristophii* | Asparagales | Amaryllidaceae | Radial | 13:16 | 04/06/2013 | Bristol | 32.2 | 29.5 | 2.7 |
| *Crinum* × *powellii* | Asparagales | Amaryllidaceae | Bilateral | 11:59 | 25/06/2014 | Bristol | 30.9 | 25 | 5.9 |
| *Galanthus nivalis* | Asparagales | Amaryllidaceae | Radial | 15:10 | 18/02/2015 | Bristol | 9.6 | 8.2 | 1.4 |
| *Narcissus hispanicus* | Asparagales | Amaryllidaceae | Radial | 15:13 | 18/02/2015 | Bristol | 10.5 | 9.4 | 1.1 |
| *Narcissus pseudonarcissus* | Asparagales | Amaryllidaceae | Radial | 10:45 | 24/03/2015 | Bristol | 12.8 | 8.1 | 4.7 |
| *Tulbaghia violacea* | Asparagales | Amaryllidaceae | Radial | 11:39 | 26/06/2014 | Bristol | 22.7 | 21.1 | 1.6 |
| *Hyacinthoides non-scripta* | Asparagales | Asparagaceae | Radial | 12:48 | 26/04/2016 | Wales | 12.4 | 10.3 | 2.1 |
| *Paradisea lusitanica* | Asparagales | Asparagaceae | Radial | 11:36 | 26/06/2014 | Bristol | 21.8 | 20.6 | 1.2 |
| *Crocosmia* 'Lucifer' | Asparagales | Iridaceae | Bilateral | 13:45 | 25/06/2014 | Bristol | 30.7 | 24.9 | 5.8 |
| *Crocus vernus* | Asparagales | Iridaceae | Radial | 14:46 | 18/02/2015 | Bristol | 13.9 | 11.7 | 2.2* |
| *Crocus* × *stellaris* | Asparagales | Iridaceae | Radial | 14:44 | 18/02/2015 | Bristol | 12.3 | 11.2 | 1.1 |
| *Cypella herbertii* | Asparagales | Iridaceae | Radial | 14:32 | 26/06/2014 | Bristol | 24 | 22.3 | 1.7 |
| *Dietes bicolor* | Asparagales | Iridaceae | Radial | 14:46 | 26/06/2014 | Bristol | 21.9 | 21.7 | 0.2 |
| *Iris sibirica* | Asparagales | Iridaceae | Bilateral | 15:28 | 09/02/2015 | Bristol | 6.3 | 6 | 0.3 |
| *Iris unguicularis* | Asparagales | Iridaceae | Bilateral | 13:25 | 04/06/2013 | Bristol | 26 | 21.3 | 4.7 |
| *Hemerocallis* 'Autumn Red' | Asparagales | Xanthorrhoeaceae | Radial | 13:47 | 25/06/2014 | Bristol | 35.5 | 26.8 | 8.7* |
| *Hemerocallis* sp. | Asparagales | Xanthorrhoeaceae | Radial | 15:54 | 26/03/2015 | Bristol | 11.1 | 10.8 | 0.3 |
| *Pasithea caerulea* | Asparagales | Xanthorrhoeaceae | Radial | 12:39 | 23/04/2016 | Wales | 21 | 20.1 | 0.9 |
| *Arctotis acaulis* | Asterales | Asteraceae | Radial | 14:36 | 26/06/2014 | Bristol | 24.7 | 22.9 | 1.8 |
| *Bellis perennis* | Asterales | Asteraceae | Radial | 13:41 | 25/06/2014 | Bristol | 29.3 | 22.3 | 7 |
| *Brachyscome iberidifolia* | Asterales | Asteraceae | Radial | 11:37 | 01/07/2016 | Wales | 27.2 | 22.4 | 4.8 |
| *Coreopsis verticillata* | Asterales | Asteraceae | Radial | 13:47 | 25/06/2014 | Bristol | 32.8 | 23.3 | 9.5 |
| *Dahlia coccinea* | Asterales | Asteraceae | Radial | 14:47 | 26/06/2014 | Bristol | 20.3 | 19.6 | 0.7 |
| *Dimorphotheca pluvialis* | Asterales | Asteraceae | Radial | 12:00 | 23/04/2016 | Wales | 26 | 19.6 | 6.4 |
| *Dimorphotheca sinuata* | Asterales | Asteraceae | Radial | 13:31 | 04/06/2013 | Bristol | 38.7 | 30.1 | 8.6 |
| *Dimorphotheca* sp. | Asterales | Asteraceae | Radial | 12:00 | 23/04/2016 | Wales | 26.8 | 19.9 | 6.9 |
| *Felicia amelloides* | Asterales | Asteraceae | Radial | 15:29 | 09/02/2015 | Bristol | 10.1 | 7.6 | 2.5 |
| *Gazania* 'Daybreak Bright Orange' | Asterales | Asteraceae | Radial | 15:45 | 01/07/2016 | Wales | 26.1 | 21.8 | 4.3* |
| *Gazania rigens* | Asterales | Asteraceae | Radial | 13:31 | 04/06/2013 | Bristol | 39.7 | 30.2 | 9.5* |
| *Gazania rigens* 'Cookie' | Asterales | Asteraceae | Radial | 12:55 | 01/07/2016 | Wales | 27.3 | 23.3 | 4 |
| *Helichrysum* sp. | Asterales | Asteraceae | Radial | 12:20 | 23/04/2016 | Wales | 24.3 | 19.8 | 4.5 |
| *Leucanthemum vulgare* | Asterales | Asteraceae | Radial | 11:24 | 04/06/2016 | Wales | 25.6 | 19.4 | 6.2 |
| *Matricaria chamomilla* | Asterales | Asteraceae | Radial | 14:59 | 25/06/2014 | Bristol | 25 | 19.9 | 5.1 |
| *Osteospermum jucundum* | Asterales | Asteraceae | Radial | 13:18 | 04/06/2013 | Bristol | 33.8 | 22 | 11.8 |
| *Rhodanthe chlorocephala* | Asterales | Asteraceae | Radial | 12:22 | 23/04/2016 | Wales | 26.6 | 20.6 | 6 |
| *Taraxacum officinale* | Asterales | Asteraceae | Radial | 14:36 | 04/05/2016 | Wales | 27 | 17.2 | 9.8 |
| *Xerochrysum bracteatum* | Asterales | Asteraceae | Radial | 12:09 | 23/04/2016 | Wales | 22 | 17.3 | 4.7 |
| *Xerochrysum bracteatum* 'Florabella Pink' | Asterales | Asteraceae | Radial | 12:21 | 23/04/2016 | Wales | 27.8 | 22 | 5.8* |
| *Zinnia peruviana* 'Red Spider' | Asterales | Asteraceae | Radial | 15:02 | 26/06/2014 | Bristol | 20.6 | 20.2 | 0.4 |
| *Campanula persicifolia* | Asterales | Campanulaceae | Radial | 15:12 | 26/06/2014 | Bristol | 19.2 | 18.7 | 0.5 |
| *Campanula portenschlagiana* | Asterales | Campanulaceae | Radial | 12:25 | 25/06/2014 | Bristol | 26.4 | 22.2 | 4.2 |
| *Campanula poscharskyana* | Asterales | Campanulaceae | Radial | 14:19 | 18/02/2015 | Bristol | 9.4 | 8.9 | 0.5 |
| *Borago officinalis* | Boraginales | Boraginaceae | Radial | 14:57 | 25/06/2014 | Bristol | 23.9 | 22.3 | 1.6 |
| *Brunnera macrophylla* | Boraginales | Boraginaceae | Radial | 11:45 | 06/05/2016 | Wales | 23.5 | 21.4 | 2.1 |
| *Arabis alpina* | Brassicales | Brassicaceae | Radial | 11:52 | 06/05/2016 | Wales | 24.3 | 23 | 1.3 |
| *Aubrieta deltoidea* | Brassicales | Brassicaceae | Radial | 11:40 | 24/03/2015 | Bristol | 12.5 | 11.3 | 1.2 |
| *Silene coronaria* | Caryophyllales | Caryophyllaceae | Radial | 15:19 | 25/06/2014 | Bristol | 24.7 | 21.9 | 2.8 |
| *Hydrangea macrophylla* | Cornales | Hydrangeaceae | Radial | 13:38 | 25/06/2014 | Bristol | 24.2 | 22 | 2.2 |
| *Hypericum calycinum* | Cornales | Hypericaceae | Radial | 15:20 | 25/06/2014 | Bristol | 28.8 | 24.5 | 4.3 |
| *Begonia coccinea* | Cucurbitales | Begoniaceae | Bilateral | 14:39 | 26/06/2014 | Bristol | 24.2 | 23.5 | 0.7 |
| *Knautia macedonica* | Disacales | Caprifoliaceae | Radial | 15:11 | 26/06/2014 | Bristol | 31.8 | 22.6 | 9.2* |
| *Knautia macedonica* 'Red Knight' | Disacales | Caprifoliaceae | Radial | 13:51 | 25/06/2014 | Bristol | 19.8 | 19.4 | 0.4 |
| *Impatiens tinctoria* | Ericales | Balsaminaceae | Bilateral | 15:08 | 26/06/2014 | Bristol | 18.6 | 16.4 | 2.2 |
| *Azalea* sp. | Ericales | Ericaceae | Bilateral | 12:01 | 06/05/2016 | Wales | 21.1 | 20 | 1.1 |
| *Rhododendron carolinianum* 'P.J. Mezitt' | Ericales | Ericaceae | Radial | 10:37 | 08/05/2016 | Wales | 27.2 | 23.5 | 3.7 |
| *Rhododendron* sp. (white) | Ericales | Ericaceae | Radial | 10:39 | 08/05/2016 | Wales | 26 | 24 | 2 |
| *Polemonium foliosissimum* | Ericales | Polemoniaceae | Radial | 11:24 | 26/06/2014 | Bristol | 19 | 18.5 | 0.5 |
| *Primula vulgaris* (mauve form) | Ericales | Primulaceae | Radial | 14:16 | 18/02/2015 | Bristol | 17.8 | 13.2 | 4.6 |
| *Primula vulgaris* (white form) | Ericales | Primulaceae | Radial | 10:14 | 08/05/2016 | Wales | 32.6 | 27.1 | 5.5* |
| *Camellia fraterna* | Ericales | Theaceae | Radial | 11:48 | 06/05/2016 | Wales | 21.6 | 20.2 | 1.4 |
| *Lotus corniculatus* | Fabales | Fabaceae | Bilateral | 12:30 | 14/05/2016 | Wales | 18.7 | 16.4 | 2.3 |
| *Mimosa pudica* | Fabales | Fabaceae | Radial | 14:42 | 26/06/2014 | Bristol | 26.6 | 26 | 0.6 |
| *Piptanthus napalensis* | Fabales | Fabaceae | Bilateral | 13:11 | 04/06/2013 | Bristol | 26 | 21.3 | 4.7 |
| *Vinca herbacea* | Gentianales | Apocynaceae | Radial | 11:51 | 25/06/2014 | Bristol | 28.2 | 23.7 | 4.5 |
| *Vinca minor* | Gentianales | Apocynaceae | Radial | 11:42 | 06/05/2016 | Wales | 28.8 | 25.1 | 3.7 |
| *Geranium albanum* | Geraniales | Geraniaceae | Radial | 11:15 | 26/03/2015 | Bristol | 19.1 | 19 | 0.1 |
| *Geranium californicum* | Geraniales | Geraniaceae | Radial | 12:25 | 23/04/2016 | Wales | 21.5 | 19.4 | 2.1 |
| *Geranium pratense* | Geraniales | Geraniaceae | Radial | 13:04 | 14/07/2016 | Wales | 25.1 | 20.7 | 4.4 |
| *Geranium procurrens* | Geraniales | Geraniaceae | Radial | 11:29 | 26/06/2014 | Bristol | 21.6 | 18.6 | 3 |
| *Geranium psilostemon* | Geraniales | Geraniaceae | Radial | 12:39 | 14/07/2016 | Wales | 29.3 | 22.3 | 7 |
| *Geranium pyrenaicum* | Geraniales | Geraniaceae | Radial | 14:32 | 25/06/2014 | Bristol | 25.3 | 23.9 | 1.4 |
| *Geranium sylvaticum* | Geraniales | Geraniaceae | Radial | 12:26 | 13/07/2016 | Wales | 23.4 | 20.2 | 3.2 |
| *Pelargonium cucullatum* | Geraniales | Geraniaceae | Bilateral | 15:53 | 26/03/2015 | Bristol | 11.1 | 10.7 | 0.4 |
| *Pelargonium echinatum* | Geraniales | Geraniaceae | Bilateral | 15:54 | 26/03/2015 | Bristol | 11 | 10.5 | 0.5 |
| *Pelargonium quercifolium* | Geraniales | Geraniaceae | Bilateral | 14:31 | 26/06/2014 | Bristol | 19.7 | 19.5 | 0.2 |
| *Acanthus hungaricus* | Lamiales | Acanthaceae | Bilateral | 11:50 | 26/06/2014 | Bristol | 24.1 | 19.5 | 4.6 |
| *Nepeta* 'Six Hills Giant' | Lamiales | Lamiaceae | Bilateral | 15:01 | 25/06/2014 | Bristol | 23.8 | 23 | 0.8 |
| *Phlomis fruticosa* | Lamiales | Lamiaceae | Bilateral | 15:10 | 25/06/2014 | Bristol | 27.1 | 26.6 | 0.5 |
| *Salvia forsskaolii* | Lamiales | Lamiaceae | Bilateral | 11:38 | 26/06/2014 | Bristol | 20.9 | 20.9 | 0 |
| *Scutellaria galericulata* | Lamiales | Lamiaceae | Bilateral | 15:08 | 25/06/2014 | Bristol | 28 | 23.8 | 4.2 |
| *Jasminum officinale* | Lamiales | Oleaceae | Radial | 14:46 | 25/06/2014 | Bristol | 28.4 | 25.1 | 3.3 |
| *Mimulus aurantiacus* | Lamiales | Phrymaceae | Bilateral | 11:45 | 26/06/2014 | Bristol | 24.1 | 23.2 | 0.9 |
| *Tulipa* 'Daydream' | Liliales | Liliaceae | Radial | 12:57 | 23/04/2016 | Wales | 19.9 | 14.6 | 5.3* |
| *Tulipa* 'Golden Appledoorn' | Liliales | Liliaceae | Radial | 12:54 | 23/04/2016 | Wales | 19.1 | 13.9 | 5.2* |
| *Tulipa hageri* 'Red Cup' | Liliales | Liliaceae | Radial | 12:57 | 23/04/2016 | Wales | 20.5 | 14.4 | 6.1 |
| *Tulipa* 'Honky Tonk' | Liliales | Liliaceae | Radial | 12:07 | 06/05/2016 | Wales | 25.7 | 22.1 | 3.6* |
| *Tulipa kaufmanniana* | Liliales | Liliaceae | Radial | 12:59 | 23/04/2016 | Wales | 17.4 | 13.1 | 4.3 |
| *Tulipa tarda* | Liliales | Liliaceae | Radial | 12:07 | 06/05/2016 | Wales | 23.8 | 21.4 | 2.4 |
| *Magnolia kobus* | Magnoliales | Magnoliaceae | Radial | 12:06 | 06/05/2016 | Wales | 23.4 | 21.2 | 2.2 |
| *Euphorbia characias* | Malpighiales | Euphorbiaceae | Radial | 15:48 | 26/03/2015 | Bristol | 6.6 | 5.6 | 1 |
| *Cistus* 'Snow Fire' | Malvales | Cistaceae | Radial | 09:32 | 03/07/2016 | Wales | 28.7 | 18 | 10.7* |
| *Cistus* 'Snow White' | Malvales | Cistaceae | Radial | 15:37 | 05/07/2016 | Wales | 25.6 | 18.3 | 7.3* |
| *Cistus* × *argenteus* | Malvales | Cistaceae | Radial | 10:46 | 23/06/2016 | Wales | 26.5 | 20 | 6.5* |
| *Cistus* × *pulverulentus* | Malvales | Cistaceae | Radial | 12:28 | 06/07/2016 | Wales | 24.1 | 21.3 | 2.8 |
| *Cistus* × *purpureus* | Malvales | Cistaceae | Radial | 16:24 | 08/06/2016 | Bristol | 30.8 | 24.4 | 6.4* |
| *Cistus* × *verguinii* | Malvales | Cistaceae | Radial | 12:31 | 23/04/2016 | Wales | 29.3 | 20.7 | 8.6* |
| *Helianthemum apenninum* | Malvales | Cistaceae | Radial | 11:52 | 26/06/2014 | Bristol | 23.5 | 19.2 | 4.3 |
| *Helianthemum nummularium* | Malvales | Cistaceae | Radial | 10:55 | 25/06/2014 | Bristol | 19.1 | 17.4 | 1.7 |
| *Alyogyne huegelii* | Malvales | Malvaceae | Radial | 12:16 | 23/04/2016 | Wales | 31 | 24.6 | 6.4 |
| *Malva sylvestris* | Malvales | Malvaceae | Radial | 10:47 | 26/06/2014 | Bristol | 19.2 | 18.6 | 0.6 |
| *Daphne odora* | Malvales | Thymelaeaceae | Radial | 14:26 | 18/02/2015 | Bristol | 11.1 | 10.7 | 0.4 |
| *Chamelaucium uncinatum* | Myrtales | Myrtaceae | Radial | 12:16 | 23/04/2016 | Wales | 24.7 | 23.1 | 1.6 |
| *Chamerion angustifolium* 'Albino' | Myrtales | Onagraceae | Radial | 11:32 | 26/06/2014 | Bristol | 19.3 | 19 | 0.3 |
| *Fuchsia magellanica* | Myrtales | Onagraceae | Radial | 14:49 | 25/06/2014 | Bristol | 27.9 | 22.8 | 5.1 |
| *Nymphaea odorata* | Nymphaeales | Nymphaeaceae | Radial | 11:57 | 26/06/2014 | Bristol | 27.5 | 20.9 | 6.6 |
| *Eschscholzia californica* | Ranunculales | Papaveraceae | Radial | 13:08 | 04/06/2013 | Bristol | 33.5 | 22.5 | 11 |
| *Papaver cambricum* | Ranunculales | Papaveraceae | Radial | 11:55 | 25/06/2014 | Bristol | 26 | 21.4 | 4.6 |
| *Papaver rhoeas* | Ranunculales | Papaveraceae | Radial | 14:56 | 26/06/2014 | Bristol | 20.4 | 19.3 | 1.1 |
| *Anemone blanda* | Ranunculales | Ranunculaceae | Radial | 15:44 | 26/03/2015 | Bristol | 20.5 | 12.3 | 8.2 |
| *Anemone nemorosa* | Ranunculales | Ranunculaceae | Radial | 12:05 | 06/05/2016 | Wales | 22.6 | 21.7 | 0.9 |
| *Aquilegia vulgaris* | Ranunculales | Ranunculaceae | Bilateral | 13:27 | 04/06/2013 | Bristol | 29.3 | 22.4 | 6.9 |
| *Ficaria verna* | Ranunculales | Ranunculaceae | Radial | 13:45 | 04/05/2016 | Wales | 23.6 | 17.2 | 6.4 |
| *Helleborus orientalis* | Ranunculales | Ranunculaceae | Radial | 15:36 | 09/02/2015 | Bristol | 7.2 | 6 | 1.2 |
| *Helleborus* × *‘*Snow White’ | Ranunculales | Ranunculaceae | Radial | 15:02 | 24/03/2015 | Bristol | 11.1 | 9 | 2.1* |
| *Nigella damascena* | Ranunculales | Ranunculaceae | Radial | 15:06 | 25/06/2014 | Bristol | 27 | 24.5 | 2.5 |
| *Ranunculus aquatilis* | Ranunculales | Ranunculaceae | Radial | 14:17 | 18/02/2015 | Bristol | 11.9 | 10.8 | 1.1 |
| *Ranunculus auricomus* | Ranunculales | Ranunculaceae | Radial | 14:25 | 25/06/2014 | Bristol | 20.7 | 19.9 | 0.8 |
| *Ranunculus bulbosus* | Ranunculales | Ranunculaceae | Radial | 09:08 | 06/05/2014 | Bristol | 19.5 | 12.3 | 7.2 |
| *Ranunculus* sp. | Ranunculales | Ranunculaceae | Radial | 12:41 | 26/04/2016 | Wales | 17.6 | 11.8 | 5.8 |
| *Trautvetteria caroliniensis* | Ranunculales | Ranunculaceae | Radial | 11:04 | 26/06/2014 | Bristol | 19.3 | 18.6 | 0.7 |
| *Chaenomeles speciosa* | Rosales | Rosaceae | Radial | 11:47 | 24/03/2015 | Bristol | 17.2 | 12.2 | 5 |
| *Exochorda* × *macrantha* | Rosales | Rosaceae | Radial | 10:20 | 08/05/2016 | Wales | 24.3 | 23.3 | 1 |
| *Fragaria vesca* | Rosales | Rosaceae | Radial | 12:00 | 06/05/2016 | Wales | 21.6 | 20.9 | 0.7 |
| *Geum chiloense* 'Lady Straheden' | Rosales | Rosaceae | Radial | 13:52 | 25/06/2014 | Bristol | 34 | 27.1 | 6.9* |
| *Geum chiloense* 'Mrs Bradshaw' | Rosales | Rosaceae | Radial | 13:45 | 25/06/2014 | Bristol | 38.3 | 30.9 | 7.4* |
| *Geum* 'Kariskaer' | Rosales | Rosaceae | Radial | 11:50 | 06/05/2016 | Wales | 25.3 | 23.3 | 2 |
| *Kerria japonica* 'Flore Pleno' | Rosales | Rosaceae | Radial | 11:56 | 06/05/2016 | Wales | 24.2 | 20.4 | 3.8 |
| *Potentilla fruticosa* 'Gibson's Scarlet' | Rosales | Rosaceae | Radial | 14:02 | 25/06/2014 | Bristol | 24.4 | 22.4 | 2 |
| *Prunus* sp. | Rosales | Rosaceae | Radial | 10:30 | 08/05/2016 | Wales | 24.3 | 23.4 | 0.9 |
| *Spiraea prunifolia* | Rosales | Rosaceae | Radial | 13:56 | 25/06/2014 | Bristol | 21.9 | 21.1 | 0.8 |
| *Paeonia peregrina* | Saxifragales | Paeoniaceae | Radial | 13:22 | 04/06/2013 | Bristol | 31.1 | 22.5 | 8.6 |
| *Bergenia cordifolia* | Saxifragales | Saxifragaceae | Radial | 14:22 | 18/02/2015 | Bristol | 16.6 | 11.6 | 5 |
| *Roscoea* sp. | Zingiberales | Zingiberaceae | Bilateral | 15:01 | 26/06/2014 | Bristol | 20.2 | 19.5 | 0.7 |
