# Supplementary material for: The diversity of floral temperature patterns, and their use by pollinators
Source: eLife. 2017 Dec 19;6:e31262. doi: 10.7554/eLife.31262 (PMC5736352; doi:10.7554/eLife.31262)
Supplement: Supplementary file 2: — All weather data was obtained from the nearest Met Office weather station: for Bristol survey days, Filton weather station (51°31'15.6"N 2°34'33.6"W); for Botanic Garden of Wales survey days, Aberporth weather station (52°07'48.0"N 4°32'20.4"W). [file elife-31262-supp2.docx]

**Supplementary File 2:** Hourly weather data is provided for each hour thermographs were collected. All weather data was obtained from the nearest Met Office weather station: for Bristol survey days, Filton weather station (51°31'15.6"N 2°34'33.6"W); for Botanic Garden of Wales survey days, Aberporth weather station (52°07'48.0"N 4°32'20.4"W).

| Date | Hour | Thermograph Sampling Location | Met Office Weather station location | Hourly Temperature (ºC) | Hourly Relative Humidity (%) | Hourly Total Cloud Cover (oktas) | Hourly Pressure at Mean Sea Level (hPa) | Hourly Rainfall  Total (mm) |
| --- | --- | --- | --- | --- | --- | --- | --- | --- |
| 04/06/2013 | 13 | Bristol | Filton | 19.7 | n/a | 0 | 1026 | 0.0 |
| 06/05/2014 | 9 | Bristol | Filton | 11.9 | 79.5 | 7 | 1007 | 0.0 |
| 25/06/2014 | 10 | Bristol | Filton | 16.9 | 51.2 | 7 | 1019 | 0.0 |
| 25/06/2014 | 11 | Bristol | Filton | 18.4 | 48.3 | 1 | 1019 | 0.0 |
| 25/06/2014 | 12 | Bristol | Filton | 18.6 | 47.8 | 0 | 1018 | 0.0 |
| 25/06/2014 | 13 | Bristol | Filton | 19.9 | 41.9 | 0 | 1018 | 0.0 |
| 25/06/2014 | 14 | Bristol | Filton | 19.8 | 41.7 | 1 | 1018 | 0.0 |
| 25/06/2014 | 15 | Bristol | Filton | 20.7 | 40.9 | 2 | 1017 | 0.0 |
| 26/06/2014 | 10 | Bristol | Filton | 18.0 | 50.3 | 4 | 1015 | 0.0 |
| 26/06/2014 | 11 | Bristol | Filton | 18.6 | 45.4 | 1 | 1014 | 0.0 |
| 26/06/2014 | 12 | Bristol | Filton | 20.2 | 44.0 | 1 | 1014 | 0.0 |
| 26/06/2014 | 13 | Bristol | Filton | 20.8 | 42.7 | 0 | 1014 | 0.0 |
| 26/06/2014 | 14 | Bristol | Filton | 19.8 | 46.5 | 5 | 1013 | 0.0 |
| 26/06/2014 | 15 | Bristol | Filton | 19.0 | 49.4 | 8 | 1013 | 0.0 |
| 09/02/2015 | 15 | Bristol | Filton | 7.2 | 68.0 | 0 | 1035 | 0.0 |
| 18/02/2015 | 14 | Bristol | Filton | 10.1 | 63.9 | 2 | 1035 | 0.0 |
| 18/02/2015 | 15 | Bristol | Filton | 10.0 | 63.9 | 7 | 1035 | 0.0 |
| 24/03/2015 | 10 | Bristol | Filton | 7.4 | 67.3 | 7 | 1012 | Trace |
| 24/03/2015 | 11 | Bristol | Filton | 7.3 | 58.9 | 5 | 1011 | 0.0 |
| 24/03/2015 | 15 | Bristol | Filton | 7.9 | 66.5 | 6 | 1010 | 0.0 |
| 26/03/2015 | 11 | Bristol | Filton | 10.6 | 71.2 | 8 | 1003 | 0.0 |
| 26/03/2015 | 15 | Bristol | Filton | 10.4 | 57.7 | 6 | 1008 | 0.0 |
| 23/04/2016 | 12 | Botanic Garden of Wales | Aberporth | 8.3 | 68.2 | 0 | 1024 | 0.0 |
| 26/04/2016 | 12 | Botanic Garden of Wales | Aberporth | 6.6 | 70.4 | 7 | 1013 | 0.0 |
| 26/04/2016 | 13 | Botanic Garden of Wales | Aberporth | 6.8 | 69.2 | 6 | 1013 | 0.0 |
| 26/04/2016 | 14 | Botanic Garden of Wales | Aberporth | 7.1 | 72.4 | 3 | 1013 | 0.0 |
| 06/05/2016 | 11 | Botanic Garden of Wales | Aberporth | 12.0 | 79.5 | 7 | 1011 | 0.0 |
| 06/05/2016 | 12 | Botanic Garden of Wales | Aberporth | 13.1 | 58.4 | 8 | 1010 | Trace |
| 08/05/2016 | 10 | Botanic Garden of Wales | Aberporth | 18.1 | 71.9 | 7 | 1004 | 0.0 |
| 14/05/2016 | 12 | Botanic Garden of Wales | Aberporth | 11.8 | 68.7 | 0 | 1022 | 0.0 |
| 04/06/2016 | 11 | Botanic Garden of Wales | Aberporth | 15.7 | 71.6 | 6 | 1018 | 0.0 |
| 08/06/2016 | 16 | Bristol | Filton | 23.3 | 63.0 | 2 | 1023 | 0.0 |
| 23/06/2016 | 10 | Botanic Garden of Wales | Aberporth | 14.7 | 71.8 | 4 | 1018 | 0.0 |
| 01/07/2016 | 11 | Botanic Garden of Wales | Aberporth | 13.3 | 70.5 | 8 | 1007 | 0.0 |
| 01/07/2016 | 12 | Botanic Garden of Wales | Aberporth | 15.1 | 63.1 | 5 | 1007 | 0.0 |
| 01/07/2016 | 15 | Botanic Garden of Wales | Aberporth | 14.7 | 68.9 | 6 | 1007 | 0.0 |
| 03/07/2016 | 9 | Botanic Garden of Wales | Aberporth | 12.8 | 90.5 | 0 | 1018 | 0.0 |
| 05/07/2016 | 15 | Botanic Garden of Wales | Aberporth | 15.1 | 63.9 | 0 | 1022 | 0.0 |
| 06/07/2016 | 12 | Botanic Garden of Wales | Aberporth | 16.7 | 68.6 | 8 | 1023 | 0.0 |
| 13/07/2016 | 12 | Botanic Garden of Wales | Aberporth | 14.1 | 71.2 | 2 | 1022 | 0.0 |
| 14/07/2016 | 12 | Botanic Garden of Wales | Aberporth | 14.3 | 75.5 | 3 | 1027 | 0.0 |
| 14/07/2016 | 13 | Botanic Garden of Wales | Aberporth | 14.5 | 75.7 | 2 | 1028 | 0.0 |
|  |  |  | **Mean** | **14.3** | **62.4** | **3.9** | **1016.8** | **0.0** |
|  |  |  | **SD** | **4.7** | **12.4** | **3.0** | **8.0** | **0** |
