# Supplementary material for: The diversity of floral temperature patterns, and their use by pollinators
Source: eLife. 2017 Dec 19;6:e31262. doi: 10.7554/eLife.31262 (PMC5736352; doi:10.7554/eLife.31262)
Supplement: Supplementary file 3: — All weather data was obtained from the nearest Met Office weather station: for Bristol survey days, Filton weather station; for Garden of Wales survey days Saron weather station (52°01'N 4°37'W) for daily temperature and rainfall data, Aberporth weather station for all other data. [file elife-31262-supp3.docx]

**Supplementary File 3:** Daily weather data for days where sampling took place. All weather data was obtained from the nearest Met Office weather station: for Bristol survey days, Filton weather station; for Garden of Wales survey days Saron weather station (52°01'N 4°37'W) for daily temperature and rainfall data, Aberporth weather station for all other data.

| Date | Thermograph Sampling Location | Met Office Weather station location | Daily Maximum Temperature (0900-0900) (ºC) | Daily Minimum Temperature (0900-0900) (ºC) | Daily Mean Temperature (0900-0900) (ºC) | Daily Total Rainfall  (0900-0900)(mm) | Daily Mean Windspeed  (0100-2400) (kn) | Daily Maximum Gust (0100 -2400) (kn) | Daily Total Sunshine(0100-2400) (hrs) |
| --- | --- | --- | --- | --- | --- | --- | --- | --- | --- |
| 04/06/2013 | Bristol | Filton | 20.8 | 8.4 | 14.6 | 0.0 | 10 | 23 | 14.9 |
| 06/05/2014 | Bristol | Filton | 16.1 | 9.5 | 12.8 | 1.8 | 10 | 21 | 6.1 |
| 25/06/2014 | Bristol | Filton | 21.5 | 12.4 | 17.0 | 0.0 | 7 | 15 | 10.0 |
| 26/06/2014 | Bristol | Filton | 21.2 | 13.8 | 17.5 | 3.2 | 7 | 21 | 1.8 |
| 09/02/2015 | Bristol | Filton | 7.8 | -4.2 | 1.8 | 0.2 | 2 | 9 | 8.7 |
| 18/02/2015 | Bristol | Filton | 10.7 | 2.7 | 6.7 | Trace | 10 | 21 | 6.3 |
| 24/03/2015 | Bristol | Filton | 8.9 | 4.0 | 6.5 | 1.8 | 5 | 20 | 4.2 |
| 26/03/2015 | Bristol | Filton | 12.1 | 3.6 | 7.9 | 0.0 | 11 | 31 | 4.6 |
| 23/04/2016 | Botanic Garden of Wales, Carmarthen | Saron/Aberporth | 12.2 | 2.4 | 7.3 | 0.0 | 9 | 21 | 10.1 |
| 26/04/2016 | Botanic Garden of Wales, Carmarthen | Saron/Aberporth | 9.1 | 1.4 | 5.3 | 4.4 | 23 | 39 | 5.1 |
| 04/05/2016 | Botanic Garden of Wales, Carmarthen | Saron/Aberporth | 14.6 | 5.3 | 10.0 | 0.0 | 12 | 24 | 11.0 |
| 06/05/2016 | Botanic Garden of Wales, Carmarthen | Saron/Aberporth | 19.9 | 4.7 | 12.3 | 0.1 | 3 | 9 | 1.1 |
| 08/05/2016 | Botanic Garden of Wales, Carmarthen | Saron/Aberporth | 24.1 | 13.0 | 18.6 | 0.0 | 14 | 33 | 6.9 |
| 14/05/2016 | Botanic Garden of Wales, Carmarthen | Saron/Aberporth | 15.0 | 8.8 | 11.9 | 0.0 | 6 | 13 | 11.4 |
| 08/06/2016 | Bristol | Filton | 24.1 | 14.8 | 19.5 | Trace | 4 | 11 | 3.6 |
| 23/06/2016 | Bristol | Filton | 20.0 | 15.4 | 17.7 | Trace | 7 | 20 | 0.5 |
| 01/07/2016 | Botanic Garden of Wales, Carmarthen | Saron/Aberporth | 16.0 | 11.7 | 13.9 | 3.3 | 18 | 34 | 5.3 |
| 03/07/2016 | Botanic Garden of Wales, Carmarthen | Saron/Aberporth | 18.7 | 9.2 | 14.0 | Trace | 8 | 17 | 15.1 |
| 05/07/2016 | Botanic Garden of Wales, Carmarthen | Saron/Aberporth | 16.3 | 11.0 | 13.7 | 0.0 | 13 | 28 | 9.7 |
| 06/07/2016 | Botanic Garden of Wales, Carmarthen | Saron/Aberporth | 17.7 | 6.2 | 12.0 | 1.2 | 10 | 21 | 1.6 |
| 13/07/2016 | Botanic Garden of Wales, Carmarthen | Saron/Aberporth | 16.8 | 9.3 | 13.1 | 3.4 | 12 | 22 | 7.7 |
|  |  | **Mean** | **16.4** | **7.8** | **12.1** | **1.1** | **9.6** | **21.6** | **6.9** |
|  |  | **SD** | **4.8** | **5.0** | **4.7** | **1.5** | **4.8** | **7.9** | **4.1** |
